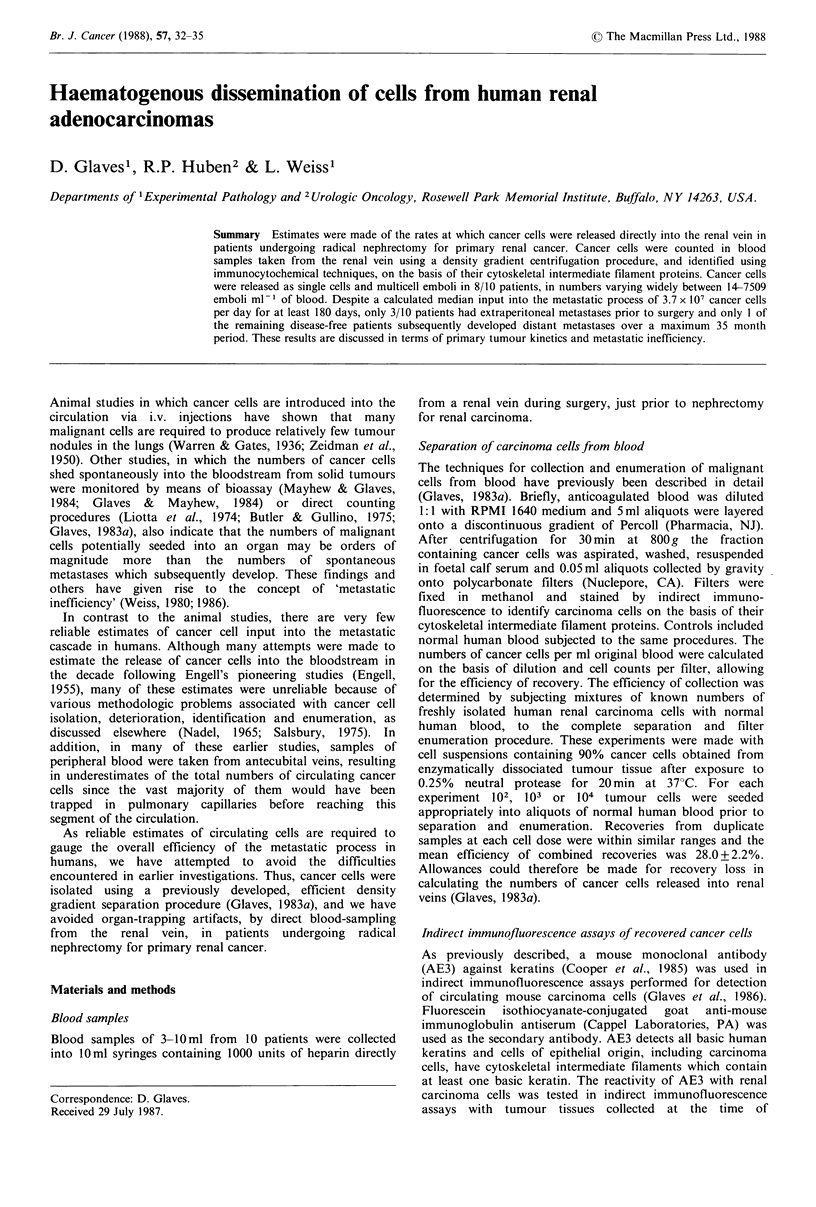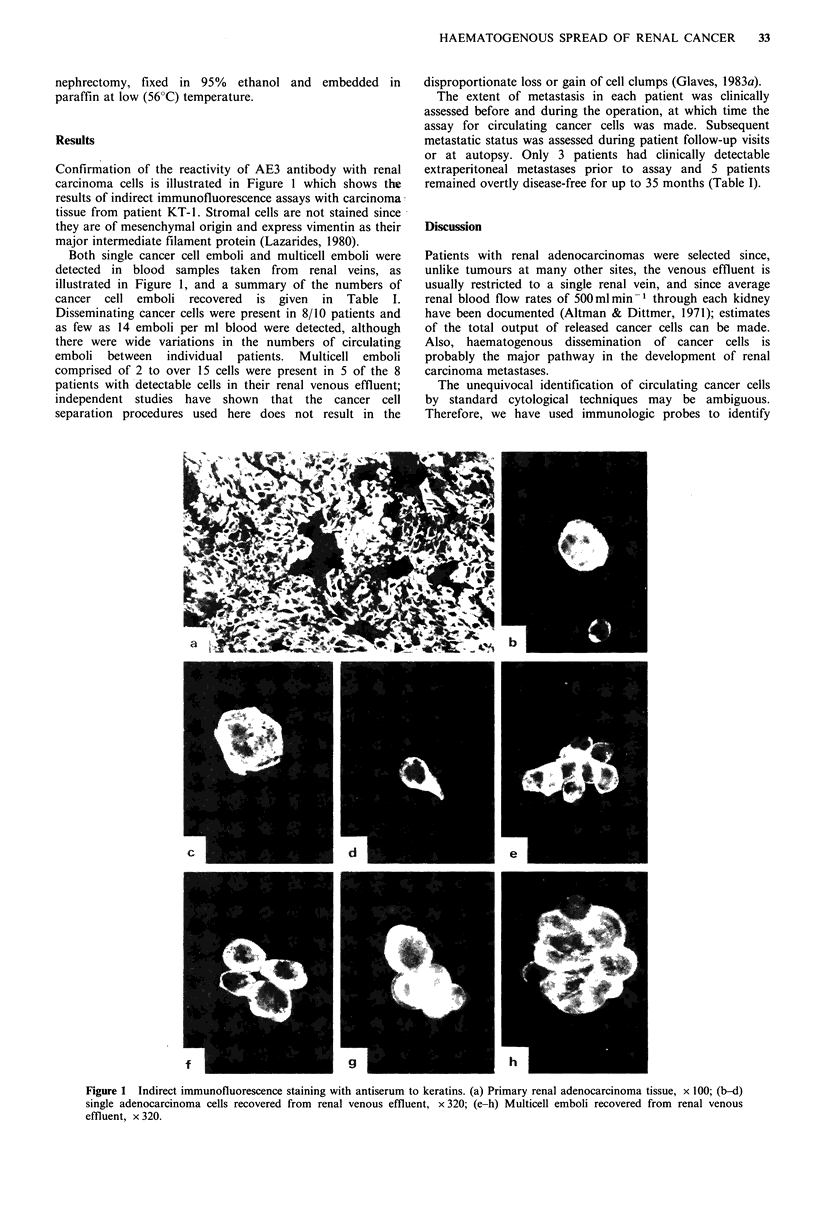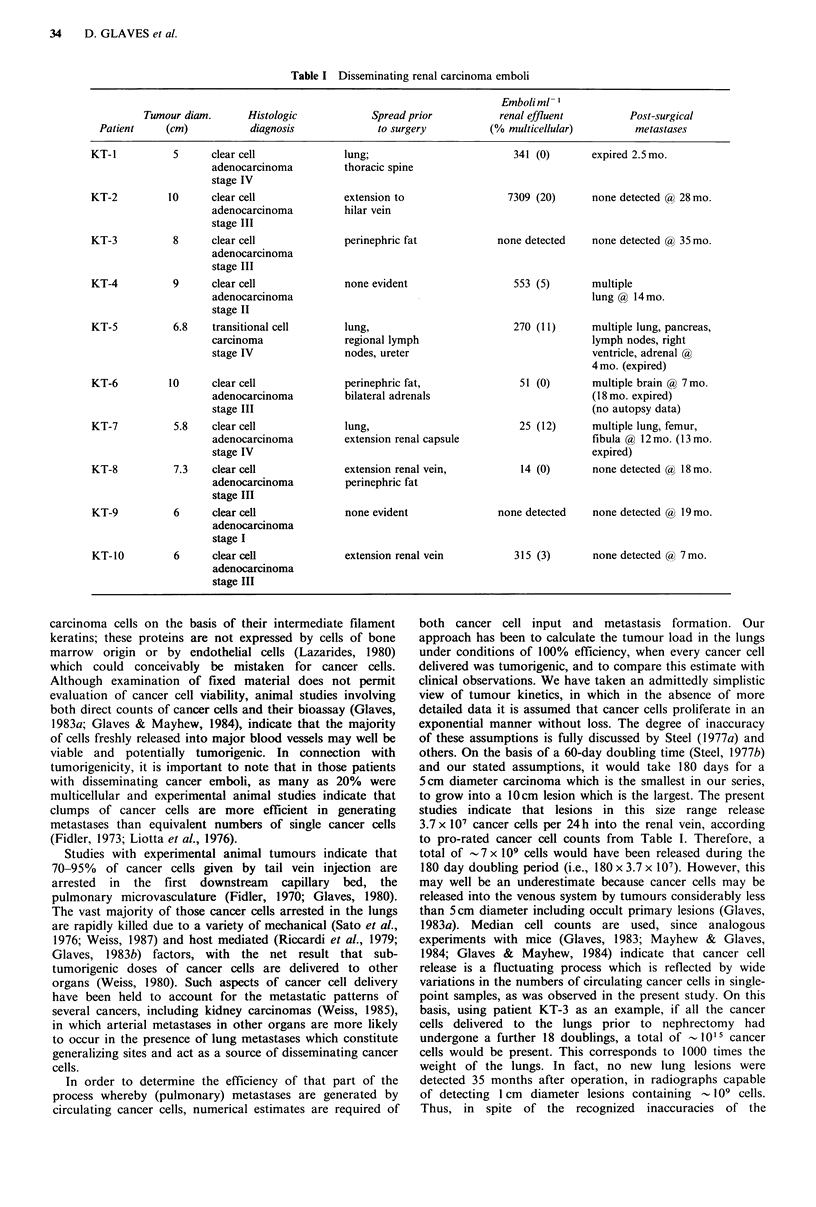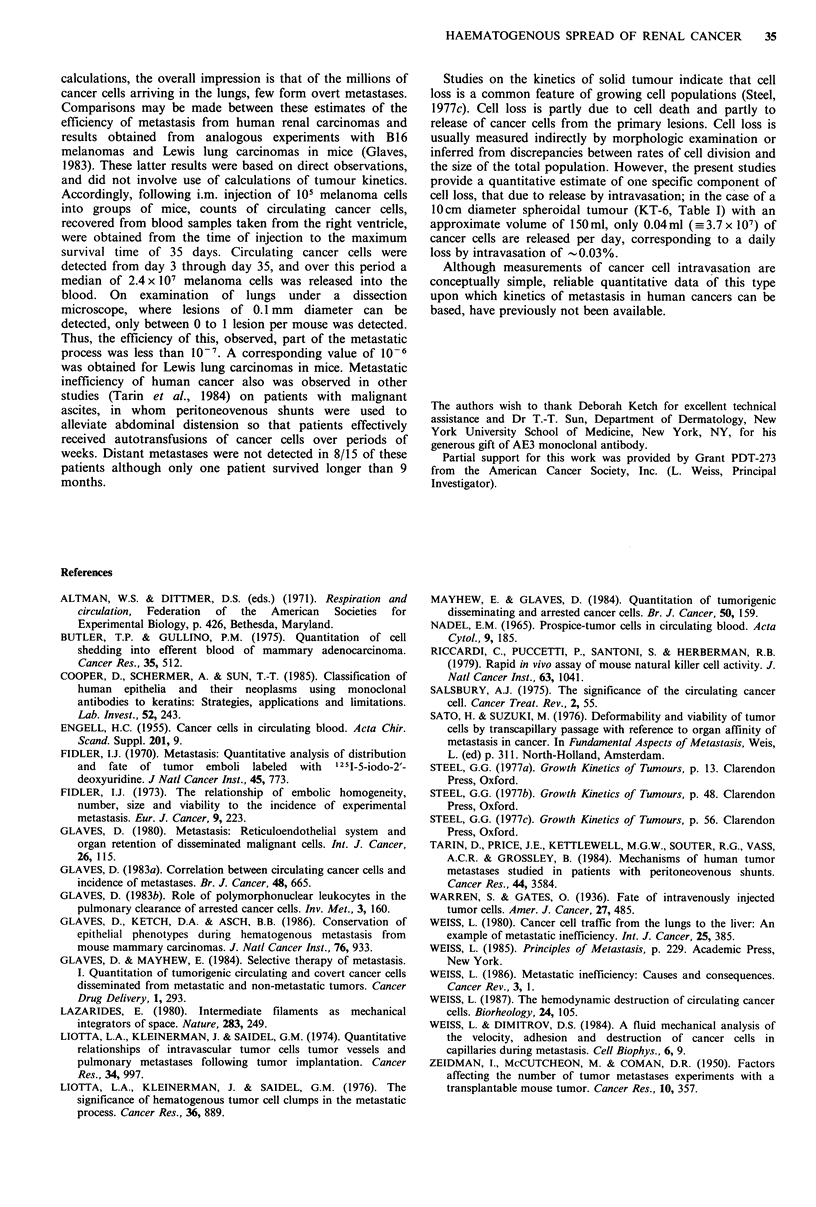# Haematogenous dissemination of cells from human renal adenocarcinomas.

**DOI:** 10.1038/bjc.1988.4

**Published:** 1988-01

**Authors:** D. Glaves, R. P. Huben, L. Weiss

**Affiliations:** Department of Experimental Pathology, Rosewell Park Memorial Institute, Buffalo, NY 14263.

## Abstract

**Images:**


Br. J. Cancer (1988), 57, 32–35 © The Macmillan Press Ltd., 1988

### Haematogenous dissemination of cells from human renal adenocarcinomas

D. Glaves[1], R.P. Huben[2] & L. Weiss[1]

*Departments of [1]Experimental Pathology and [2]Urologic Oncology, Rosewell Park Memorial Institute, Buffalo, NY 14263, USA.*

**Summary** Estimates were made of the rates at which cancer cells were released directly into the renal vein in patients undergoing radical nephrectomy for primary renal cancer. Cancer cells were counted in blood samples taken from the renal vein using a density gradient centrifugation procedure, and identified using immunocytochemical techniques, on the basis of their cytoskeletal intermediate filament proteins. Cancer cells were released as single cells and multicell emboli in 8/10 patients, in numbers varying widely between 14–7509 emboli $ml^{-1}$ of blood. Despite a calculated median input into the metastatic process of $3.7 \times 10^7$ cancer cells per day for at least 180 days, only 3/10 patients had extraperitoneal metastases prior to surgery and only 1 of the remaining disease-free patients subsequently developed distant metastases over a maximum 35 month period. These results are discussed in terms of primary tumour kinetics and metastatic inefficiency.

Animal studies in which cancer cells are introduced into the circulation via i.v. injections have shown that many malignant cells are required to produce relatively few tumour nodules in the lungs (Warren & Gates, 1936; Zeidman *et al.*, 1950). Other studies, in which the numbers of cancer cells shed spontaneously into the bloodstream from solid tumours were monitored by means of bioassay (Mayhew & Glaves, 1984; Glaves & Mayhew, 1984) or direct counting procedures (Liotta *et al.*, 1974; Butler & Gullino, 1975; Glaves, 1983*a*), also indicate that the numbers of malignant cells potentially seeded into an organ may be orders of magnitude more than the numbers of spontaneous metastases which subsequently develop. These findings and others have given rise to the concept of 'metastatic inefficiency' (Weiss, 1980; 1986).

In contrast to the animal studies, there are very few reliable estimates of cancer cell input into the metastatic cascade in humans. Although many attempts were made to estimate the release of cancer cells into the bloodstream in the decade following Engell's pioneering studies (Engell, 1955), many of these estimates were unreliable because of various methodologic problems associated with cancer cell isolation, deterioration, identification and enumeration, as discussed elsewhere (Nadel, 1965; Salsbury, 1975). In addition, in many of these earlier studies, samples of peripheral blood were taken from antecubital veins, resulting in underestimates of the total numbers of circulating cancer cells since the vast majority of them would have been trapped in pulmonary capillaries before reaching this segment of the circulation.

As reliable estimates of circulating cells are required to gauge the overall efficiency of the metastatic process in humans, we have attempted to avoid the difficulties encountered in earlier investigations. Thus, cancer cells were isolated using a previously developed, efficient density gradient separation procedure (Glaves, 1983*a*), and we have avoided organ-trapping artifacts, by direct blood-sampling from the renal vein, in patients undergoing radical nephrectomy for primary renal cancer.

### Materials and methods

### *Blood samples*

Blood samples of 3–10 ml from 10 patients were collected into 10 ml syringes containing 1000 units of heparin directly from a renal vein during surgery, just prior to nephrectomy for renal carcinoma.

### *Separation of carcinoma cells from blood*

The techniques for collection and enumeration of malignant cells from blood have previously been described in detail (Glaves, 1983*a*). Briefly, anticoagulated blood was diluted 1:1 with RPMI 1640 medium and 5 ml aliquots were layered onto a discontinuous gradient of Percoll (Pharmacia, NJ). After centrifugation for 30 min at 800 *g* the fraction containing cancer cells was aspirated, washed, resuspended in foetal calf serum and 0.05 ml aliquots collected by gravity onto polycarbonate filters (Nuclepore, CA). Filters were fixed in methanol and stained by indirect immunofluorescence to identify carcinoma cells on the basis of their cytoskeletal intermediate filament proteins. Controls included normal human blood subjected to the same procedures. The numbers of cancer cells per ml original blood were calculated on the basis of dilution and cell counts per filter, allowing for the efficiency of recovery. The efficiency of collection was determined by subjecting mixtures of known numbers of freshly isolated human renal carcinoma cells with normal human blood, to the complete separation and filter enumeration procedure. These experiments were made with cell suspensions containing 90% cancer cells obtained from enzymatically dissociated tumour tissue after exposure to 0.25% neutral protease for 20 min at 37°C. For each experiment $10^2$, $10^3$ or $10^4$ tumour cells were seeded appropriately into aliquots of normal human blood prior to separation and enumeration. Recoveries from duplicate samples at each cell dose were within similar ranges and the mean efficiency of combined recoveries was $28.0 \pm 2.2\%$. Allowances could therefore be made for recovery loss in calculating the numbers of cancer cells released into renal veins (Glaves, 1983*a*).

### *Indirect immunofluorescence assays of recovered cancer cells*

As previously described, a mouse monoclonal antibody (AE3) against keratins (Cooper *et al.*, 1985) was used in indirect immunofluorescence assays performed for detection of circulating mouse carcinoma cells (Glaves *et al.*, 1986). Fluorescein isothiocyanate-conjugated goat anti-mouse immunoglobulin antiserum (Cappel Laboratories, PA) was used as the secondary antibody. AE3 detects all basic human keratins and cells of epithelial origin, including carcinoma cells, have cytoskeletal intermediate filaments which contain at least one basic keratin. The reactivity of AE3 with renal carcinoma cells was tested in indirect immunofluorescence assays with tumour tissues collected at the time of

Correspondence: D. Glaves.
Received 29 July 1987.

nephrectomy, fixed in 95% ethanol and embedded in paraffin at low (56°C) temperature.

### Results

Confirmation of the reactivity of AE3 antibody with renal carcinoma cells is illustrated in Figure 1 which shows the results of indirect immunofluorescence assays with carcinoma tissue from patient KT-1. Stromal cells are not stained since they are of mesenchymal origin and express vimentin as their major intermediate filament protein (Lazarides, 1980).

Both single cancer cell emboli and multicell emboli were detected in blood samples taken from renal veins, as illustrated in Figure 1, and a summary of the numbers of cancer cell emboli recovered is given in Table I. Disseminating cancer cells were present in 8/10 patients and as few as 14 emboli per ml blood were detected, although there were wide variations in the numbers of circulating emboli between individual patients. Multicell emboli comprised of 2 to over 15 cells were present in 5 of the 8 patients with detectable cells in their renal venous effluent; independent studies have shown that the cancer cell separation procedures used here does not result in the disproportionate loss or gain of cell clumps (Glaves, 1983*a*).

The extent of metastasis in each patient was clinically assessed before and during the operation, at which time the assay for circulating cancer cells was made. Subsequent metastatic status was assessed during patient follow-up visits or at autopsy. Only 3 patients had clinically detectable extraperitoneal metastases prior to assay and 5 patients remained overtly disease-free for up to 35 months (Table I).

### Discussion

Patients with renal adenocarcinomas were selected since, unlike tumours at many other sites, the venous effluent is usually restricted to a single renal vein, and since average renal blood flow rates of 500 ml min$^{-1}$ through each kidney have been documented (Altman & Dittmer, 1971); estimates of the total output of released cancer cells can be made. Also, haematogenous dissemination of cancer cells is probably the major pathway in the development of renal carcinoma metastases.

The unequivocal identification of circulating cancer cells by standard cytological techniques may be ambiguous. Therefore, we have used immunologic probes to identify

**Figure 1** Indirect immunofluorescence staining with antiserum to keratins. (a) Primary renal adenocarcinoma tissue, ×100; (b–d) single adenocarcinoma cells recovered from renal venous effluent, ×320; (e–h) Multicell emboli recovered from renal venous effluent, ×320.

**Table I** Disseminating renal carcinoma emboli

| *Patient* | *Tumour diam. (cm)* | *Histologic diagnosis* | *Spread prior to surgery* | *Emboli ml⁻¹ renal effluent (% multicellular)* | *Post-surgical metastases* |
|---|---|---|---|---|---|
| KT-1 | 5 | clear cell adenocarcinoma stage IV | lung; thoracic spine | 341 (0) | expired 2.5 mo. |
| KT-2 | 10 | clear cell adenocarcinoma stage III | extension to hilar vein | 7309 (20) | none detected @ 28 mo. |
| KT-3 | 8 | clear cell adenocarcinoma stage III | perinephric fat | none detected | none detected @ 35 mo. |
| KT-4 | 9 | clear cell adenocarcinoma stage II | none evident | 553 (5) | multiple lung @ 14 mo. |
| KT-5 | 6.8 | transitional cell carcinoma stage IV | lung, regional lymph nodes, ureter | 270 (11) | multiple lung, pancreas, lymph nodes, right ventricle, adrenal @ 4 mo. (expired) |
| KT-6 | 10 | clear cell adenocarcinoma stage III | perinephric fat, bilateral adrenals | 51 (0) | multiple brain @ 7 mo. (18 mo. expired) (no autopsy data) |
| KT-7 | 5.8 | clear cell adenocarcinoma stage IV | lung, extension renal capsule | 25 (12) | multiple lung, femur, fibula @ 12 mo. (13 mo. expired) |
| KT-8 | 7.3 | clear cell adenocarcinoma stage III | extension renal vein, perinephric fat | 14 (0) | none detected @ 18 mo. |
| KT-9 | 6 | clear cell adenocarcinoma stage I | none evident | none detected | none detected @ 19 mo. |
| KT-10 | 6 | clear cell adenocarcinoma stage III | extension renal vein | 315 (3) | none detected @ 7 mo. |

carcinoma cells on the basis of their intermediate filament keratins; these proteins are not expressed by cells of bone marrow origin or by endothelial cells (Lazarides, 1980) which could conceivably be mistaken for cancer cells. Although examination of fixed material does not permit evaluation of cancer cell viability, animal studies involving both direct counts of cancer cells and their bioassay (Glaves, 1983*a*; Glaves & Mayhew, 1984), indicate that the majority of cells freshly released into major blood vessels may well be viable and potentially tumorigenic. In connection with tumorigenicity, it is important to note that in those patients with disseminating cancer emboli, as many as 20% were multicellular and experimental animal studies indicate that clumps of cancer cells are more efficient in generating metastases than equivalent numbers of single cancer cells (Fidler, 1973; Liotta *et al.*, 1976).

Studies with experimental animal tumours indicate that 70–95% of cancer cells given by tail vein injection are arrested in the first downstream capillary bed, the pulmonary microvasculature (Fidler, 1970; Glaves, 1980). The vast majority of those cancer cells arrested in the lungs are rapidly killed due to a variety of mechanical (Sato *et al.*, 1976; Weiss, 1987) and host mediated (Riccardi *et al.*, 1979; Glaves, 1983*b*) factors, with the net result that sub-tumorigenic doses of cancer cells are delivered to other organs (Weiss, 1980). Such aspects of cancer cell delivery have been held to account for the metastatic patterns of several cancers, including kidney carcinomas (Weiss, 1985), in which arterial metastases in other organs are more likely to occur in the presence of lung metastases which constitute generalizing sites and act as a source of disseminating cancer cells.

In order to determine the efficiency of that part of the process whereby (pulmonary) metastases are generated by circulating cancer cells, numerical estimates are required of both cancer cell input and metastasis formation. Our approach has been to calculate the tumour load in the lungs under conditions of 100% efficiency, when every cancer cell delivered was tumorigenic, and to compare this estimate with clinical observations. We have taken an admittedly simplistic view of tumour kinetics, in which in the absence of more detailed data it is assumed that cancer cells proliferate in an exponential manner without loss. The degree of inaccuracy of these assumptions is fully discussed by Steel (1977*a*) and others. On the basis of a 60-day doubling time (Steel, 1977*b*) and our stated assumptions, it would take 180 days for a 5 cm diameter carcinoma which is the smallest in our series, to grow into a 10 cm lesion which is the largest. The present studies indicate that lesions in this size range release $3.7 \times 10^7$ cancer cells per 24 h into the renal vein, according to pro-rated cancer cell counts from Table I. Therefore, a total of $\sim 7 \times 10^9$ cells would have been released during the 180 day doubling period (i.e., $180 \times 3.7 \times 10^7$). However, this may well be an underestimate because cancer cells may be released into the venous system by tumours considerably less than 5 cm diameter including occult primary lesions (Glaves, 1983*a*). Median cell counts are used, since analogous experiments with mice (Glaves, 1983; Mayhew & Glaves, 1984; Glaves & Mayhew, 1984) indicate that cancer cell release is a fluctuating process which is reflected by wide variations in the numbers of circulating cancer cells in single-point samples, as was observed in the present study. On this basis, using patient KT-3 as an example, if all the cancer cells delivered to the lungs prior to nephrectomy had undergone a further 18 doublings, a total of $\sim 10^{15}$ cancer cells would be present. This corresponds to 1000 times the weight of the lungs. In fact, no new lung lesions were detected 35 months after operation, in radiographs capable of detecting 1 cm diameter lesions containing $\sim 10^9$ cells. Thus, in spite of the recognized inaccuracies of the calculations, the overall impression is that of the millions of cancer cells arriving in the lungs, few form overt metastases. Comparisons may be made between these estimates of the efficiency of metastasis from human renal carcinomas and results obtained from analogous experiments with B16 melanomas and Lewis lung carcinomas in mice (Glaves, 1983). These latter results were based on direct observations, and did not involve use of calculations of tumour kinetics. Accordingly, following i.m. injection of $10^5$ melanoma cells into groups of mice, counts of circulating cancer cells, recovered from blood samples taken from the right ventricle, were obtained from the time of injection to the maximum survival time of 35 days. Circulating cancer cells were detected from day 3 through day 35, and over this period a median of $2.4 \times 10^7$ melanoma cells was released into the blood. On examination of lungs under a dissection microscope, where lesions of 0.1 mm diameter can be detected, only between 0 to 1 lesion per mouse was detected. Thus, the efficiency of this, observed, part of the metastatic process was less than $10^{-7}$. A corresponding value of $10^{-6}$ was obtained for Lewis lung carcinomas in mice. Metastatic inefficiency of human cancer also was observed in other studies (Tarin *et al.*, 1984) on patients with malignant ascites, in whom peritoneovenous shunts were used to alleviate abdominal distension so that patients effectively received autotransfusions of cancer cells over periods of weeks. Distant metastases were not detected in 8/15 of these patients although only one patient survived longer than 9 months.

Studies on the kinetics of solid tumour indicate that cell loss is a common feature of growing cell populations (Steel, 1977*c*). Cell loss is partly due to cell death and partly to release of cancer cells from the primary lesions. Cell loss is usually measured indirectly by morphologic examination or inferred from discrepancies between rates of cell division and the size of the total population. However, the present studies provide a quantitative estimate of one specific component of cell loss, that due to release by intravasation; in the case of a 10 cm diameter spheroidal tumour (KT-6, Table I) with an approximate volume of 150 ml, only 0.04 ml ($\equiv 3.7 \times 10^7$) of cancer cells are released per day, corresponding to a daily loss by intravasation of $\sim 0.03\%$.

Although measurements of cancer cell intravasation are conceptually simple, reliable quantitative data of this type upon which kinetics of metastasis in human cancers can be based, have previously not been available.

The authors wish to thank Deborah Ketch for excellent technical assistance and Dr T.-T. Sun, Department of Dermatology, New York University School of Medicine, New York, NY, for his generous gift of AE3 monoclonal antibody.

Partial support for this work was provided by Grant PDT-273 from the American Cancer Society, Inc. (L. Weiss, Principal Investigator).

### References

ALTMAN, W.S. & DITTMER, D.S. (eds.) (1971). *Respiration and circulation*, Federation of the American Societies for Experimental Biology, p. 426, Bethesda, Maryland.

BUTLER, T.P. & GULLINO, P.M. (1975). Quantitation of cell shedding into efferent blood of mammary adenocarcinoma. *Cancer Res.*, **35**, 512.

COOPER, D., SCHERMER, A. & SUN, T.-T. (1985). Classification of human epithelia and their neoplasms using monoclonal antibodies to keratins: Strategies, applications and limitations. *Lab. Invest.*, **52**, 243.

ENGELL, H.C. (1955). Cancer cells in circulating blood. *Acta Chir. Scand.* Suppl. **201**, 9.

FIDLER, I.J. (1970). Metastasis: Quantitative analysis of distribution and fate of tumor emboli labeled with $^{125}$I-5-iodo-2′-deoxyuridine. *J Natl Cancer Inst.*, **45**, 773.

FIDLER, I.J. (1973). The relationship of embolic homogeneity, number, size and viability to the incidence of experimental metastasis. *Eur. J. Cancer*, **9**, 223.

GLAVES, D. (1980). Metastasis: Reticuloendothelial system and organ retention of disseminated malignant cells. *Int. J. Cancer*, **26**, 115.

GLAVES, D. (1983*a*). Correlation between circulating cancer cells and incidence of metastases. *Br. J. Cancer*, **48**, 665.

GLAVES, D. (1983*b*). Role of polymorphonuclear leukocytes in the pulmonary clearance of arrested cancer cells. *Inv. Met.*, **3**, 160.

GLAVES, D., KETCH, D.A. & ASCH, B.B. (1986). Conservation of epithelial phenotypes during hematogenous metastasis from mouse mammary carcinomas. *J. Natl Cancer Inst.*, **76**, 933.

GLAVES, D. & MAYHEW, E. (1984). Selective therapy of metastasis. I. Quantitation of tumorigenic circulating and covert cancer cells disseminated from metastatic and non-metastatic tumors. *Cancer Drug Delivery*, **1**, 293.

LAZARIDES, E. (1980). Intermediate filaments as mechanical integrators of space. *Nature*, **283**, 249.

LIOTTA, L.A., KLEINERMAN, J. & SAIDEL, G.M. (1974). Quantitative relationships of intravascular tumor cells tumor vessels and pulmonary metastases following tumor implantation. *Cancer Res.*, **34**, 997.

LIOTTA, L.A., KLEINERMAN, J. & SAIDEL, G.M. (1976). The significance of hematogenous tumor cell clumps in the metastatic process. *Cancer Res.*, **36**, 889.

MAYHEW, E. & GLAVES, D. (1984). Quantitation of tumorigenic disseminating and arrested cancer cells. *Br. J. Cancer*, **50**, 159.

NADEL, E.M. (1965). Prospice-tumor cells in circulating blood. *Acta Cytol.*, **9**, 185.

RICCARDI, C., PUCCETTI, P., SANTONI, S. & HERBERMAN, R.B. (1979). Rapid *in vivo* assay of mouse natural killer cell activity. *J. Natl Cancer Inst.*, **63**, 1041.

SALSBURY, A.J. (1975). The significance of the circulating cancer cell. *Cancer Treat. Rev.*, **2**, 55.

SATO, H. & SUZUKI, M. (1976). Deformability and viability of tumor cells by transcapillary passage with reference to organ affinity of metastasis in cancer. In *Fundamental Aspects of Metastasis*, Weis, L. (ed) p. 311. North-Holland, Amsterdam.

STEEL, G.G. (1977*a*). *Growth Kinetics of Tumours*, p. 13. Clarendon Press, Oxford.

STEEL, G.G. (1977*b*). *Growth Kinetics of Tumours*, p. 48. Clarendon Press, Oxford.

STEEL, G.G. (1977*c*). *Growth Kinetics of Tumours*, p. 56. Clarendon Press, Oxford.

TARIN, D., PRICE, J.E., KETTLEWELL, M.G.W., SOUTER, R.G., VASS, A.C.R. & GROSSLEY, B. (1984). Mechanisms of human tumor metastases studied in patients with peritoneovenous shunts. *Cancer Res.*, **44**, 3584.

WARREN, S. & GATES, O. (1936). Fate of intravenously injected tumor cells. *Amer. J. Cancer*, **27**, 485.

WEISS, L. (1980). Cancer cell traffic from the lungs to the liver: An example of metastatic inefficiency. *Int. J. Cancer*, **25**, 385.

WEISS, L. (1985). *Principles of Metastasis*, p. 229. Academic Press, New York.

WEISS, L. (1986). Metastatic inefficiency: Causes and consequences. *Cancer Rev.*, **3**, 1.

WEISS, L. (1987). The hemodynamic destruction of circulating cancer cells. *Biorheology*, **24**, 105.

WEISS, L. & DIMITROV, D.S. (1984). A fluid mechanical analysis of the velocity, adhesion and destruction of cancer cells in capillaries during metastasis. *Cell Biophys.*, **6**, 9.

ZEIDMAN, I., McCUTCHEON, M. & COMAN, D.R. (1950). Factors affecting the number of tumor metastases experiments with a transplantable mouse tumor. *Cancer Res.*, **10**, 357.